# The impact of obesity on male fecundity: a Brazilian study

**DOI:** 10.5935/1518-0557.20160031

**Published:** 2016

**Authors:** Victor T. Dubeux, Thaisa Renovato, Ana Clara Esteves, Lialyz André, Aylana de Oliveira, Ivan Araújo Penna

**Affiliations:** 1 Division of Infertility and Reproductive Medicine, Department of Mother and Child Health. Fluminense Federal University, Niterói, RJ, Brazil.

**Keywords:** Obesity, sperm count, infertility

## Abstract

**Introduction:**

Obesity has become a major problem in most developed countries. Infertility ranks high among the issues brought by excessive weight gain, particularly as it may affect male sexual function. This study investigated a population of Brazilian infertile men in an attempt to establish a relationship between obesity and infertility.

**Methods:**

A total of 153 infertile men had their anthropometric data collected and were divided into groups according to their body mass index and waist circumference measurements. Sperm parameters including sperm count, concentration, morphology, and motility were compared.

**Results:**

Multivariate analysis failed to show a positive correlation between excessive weight gain or increased waist circumference, and sperm alterations in a population of infertile men.

**Conclusions:**

The findings described in this study support the idea that there is no association between obesity and semen alterations in a population of infertile men.

## INTRODUCTION

The World Health Organization (WHO) defines infertility as the inability to achieve pregnancy within 12 months of regular intercourses for couples in conception. Infertility is estimated to affect the lives of at least 10% of the global population (Cooper *et al*., 2010). The male factor alone accounts for approximately 25-30% of the cases of infertility (Taylor *et al.*, 2003).

Meanwhile, most developed nations have reported increases in the numbers of overweight and obese men and women in their reproductive years, in addition to more cases of diseases related to obesity such as hypertension, diabetes, and vascular disorders (Hammoud *et al*., 2008).

Weight gain may be associated to changes in the reproductive system, including hormonal alterations due to the impact on testosterone production (Pasquali *et al*., 2007; Qin *et al*., 2007; Pasquali, 2006). However, direct effects on semen parameters are yet to be proven (MacDonald *et al*., 2007; Jensen *et al*., 2008).

This study aimed to investigate the effects of excessive weight gain on the semen parameters of a population of Brazilian infertile men.

## MATERIALS AND METHODS

All male individuals referred to our department between September 2014 and September 2015 due to complaints of infertility were assessed before joining the study. Study participants gave written consent before joining the study, and their identities were anonymized. Only individuals aged 18 years or older who failed to conceive for at least a year while off contraceptive methods, as per the 2010 WHO definition of infertility (Cooper *et al*., 2010), were enrolled in the study. Subjects with genital alterations that could lead to subfertility - such as vasectomy, presence of varicocele, impalpable vas deferens, urethral strictures, ectopic or atrophic testis - and individuals on drugs and smokers were excluded from the study.

Anthropometric data were obtained on the same day semen samples were collected and processed. All data were measured twice and a peer made a third measurement whenever significant differences were observed. Height was measured in centimeters with the patients standing with their backs against a wall and no shoes on. Weight was measured in kilograms with the patients wearing only underwear and standing with their feet together on a scale; all patients were weighed on the same scale. The body mass index (BMI) was calculated as weight in kilograms divided by the squared height in meters.

Study participants were divided into groups based on their BMIs. Group 1 - Normal (BMI < 25); Group 2 - Overweight (25.0<BMI<30); and Group 3 - Obese (BMI>30.0)

Waist Circumference (WC) was measured in centimeters at the end of a normal exhalation. The tape measure was positioned at the midpoint between the 12th rib and the iliac crest.

The patients were then divided into two groups, one with WC ≤ 102cm and one with WC > 102cm (cutoff-points for cardiologic risk according to the WHO).

Sterile containers were used to hold the semen samples collected in the morning period by masturbation after three to five days of abstinence. The samples were sent for analysis immediately after collection and were processed at our facilities by the same technician, according to the World Health Organization 2010 criteria for semen examination (volume 1.5 ml; sperm count 15x106/ml; total sperm count 39x106; motility 32% A+B; and morphology 4% Kruger).

### Statistical analysis

A sample size of 153 patients was deemed sufficient to identify a Spearman's RHO of at least 0.25 between spermatozoa concentration and BMI, with a 0.95 confidence interval and a statistical power of 0.8.

Continuous variables were described in terms of median values (interquartile range) and categorical variables as counts (ratios). The associations between continuous variables were tested with Spearman's rank correlation coefficient. The associations between categorical variables were tested with Fisher's exact and Chi-square tests.

The Mann-Whitney and Kruskal-Wallis tests were used to assess the distribution of continuous variables among different levels of categorical variables. A significance level of 0.05 was adopted for all statistical tests; the data sets were processed on Stata® version 14.0 (2015. StataCorp. College Station, TX, USA)

## RESULTS

Table 1 shows the distribution of patients based on WC and BMI measurements. Since only two patients were underweight, they were deemed normal and added to the group with a normal BMI.

**Table 1:** Patient BMI and WC distributions

	Patients	Percentage
Waist Circumference		
<102cm	119	75.32%
>102cm	39	24.68%
Total	158	100%
		
Body Mass Index		
Normal	55	34.81%
Overweight	56	35.44%
Obese	47	29.75%
Total	158	100%

**Figure 1:**
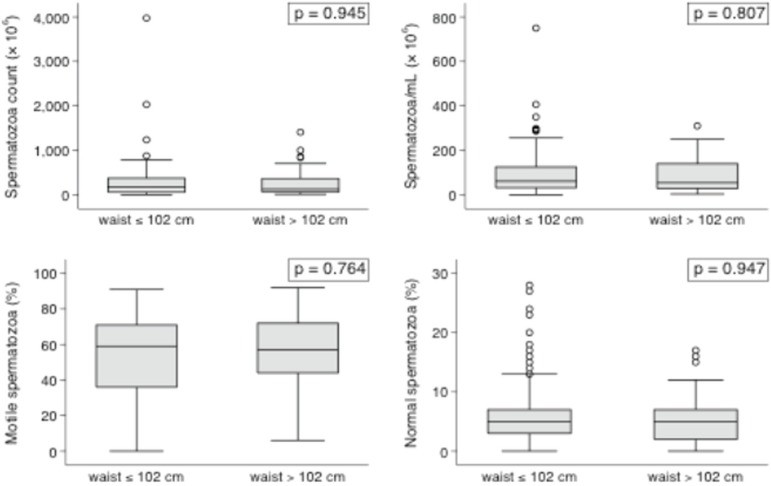
Box plot representation of sperm parameters according to WC.

**Figure 2:**
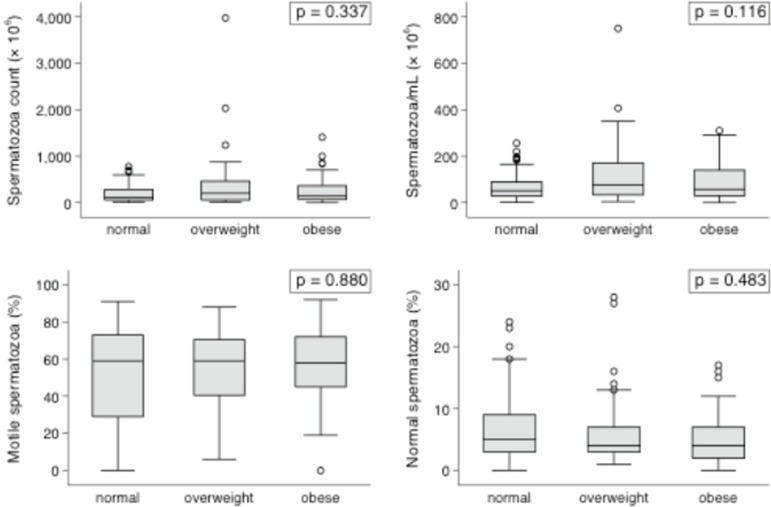
Box plot representation of sperm parameters according to the BMI.

Multifactorial analysis failed to show any impact from BMI or WC measurements upon the studied variables when obese and non-obese individuals were compared. The continuous variables shown in the box plots (Figures 1 and 2) had interquartile ranges and medians with insignificant *P*-values in all categories for BMI and WC comparisons.

**Figure 3:**
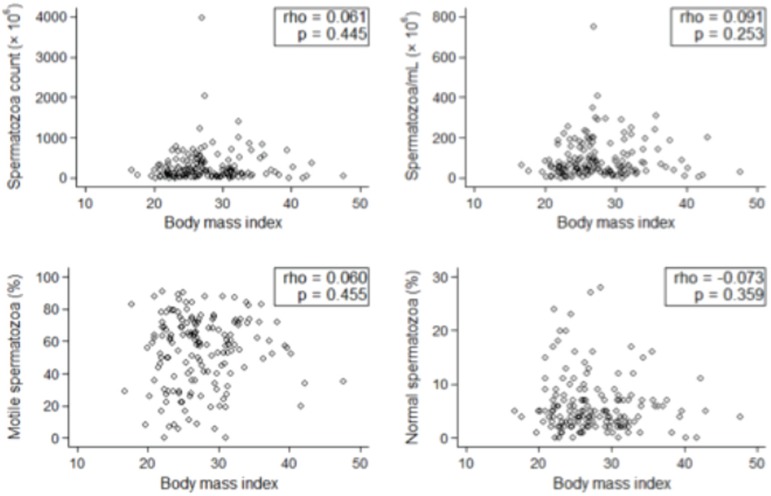
Patient BMI distribution

**Figure 4:**
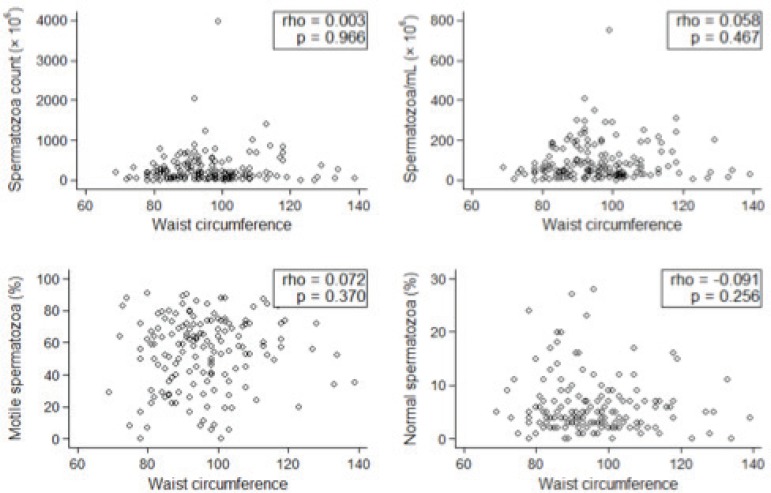
Patient WC distribution

In regards to continuous variables, the patient distribution also revealed an absence of association between obesity and sperm parameters (Figures 3 and 4).

This study failed to show a statistically significant *P*-value in the relationship between obesity and sperm parameters.

## DISCUSSION

To our knowledge, this has been the first study carried out in Brazil to look into the relationship between obesity and semen parameters.

There are two ways to assess a man's degree of obesity. One is the BMI, an indicator of whole-body fat distribution calculated from one's height and weight. The BMI is generally used to assess the distribution of fat throughout the body surface, particularly the deposition of fat in peripheral tissues. The other is the WC, which reflects visceral fat deposits directly related to the risk of cardiovascular disease (Hammoud *et al*., 2008)

The BMI distribution in our population was fairly even, but greater numbers of individuals with a WC < 102 cm were observed. The WC was also considered a valid parameter of obesity in this study, since it has been predominantly used to assess the risk cardiovascular disease.

Several reports on the effects of obesity on infertility were published in the last decade (MacDonald *et al*., 2010; Jensen *et al.*, 2008; Kupka *et al*., 2011). It has been found that male weight gain might lead to hormonal disorders affecting sex hormone-binding globulin and decreasing testosterone levels. Since free testosterone and follicular stimulating hormone bear direct impact on spermatogenesis, and once both hormones are slightly affected by obesity, it is plausible to think that semen parameters may be altered in this subgroup of patients (MacDonald *et al*., 2010; Håkonsen *et al.*, 2011; Teerds *et al*., 2011).

The hormonal status of our patients was not assessed because, as mentioned above, obese men are at a higher risk of presenting these alterations. This analysis aimed to assess the influence of obesity on male fecundity in infertile Brazilian men.

Although some authors have found male overweight to have no impact on sperm motility (Jensen *et al*., 2004; Aggerholm *et al*., 2008; Chavarro *et al*., 2010; Fejes *et al.*, 2006; Pauli *et al*., 2008), morphology (Jensen *et al.*, 2004; Chavarro *et al*., 2010; Fejes *et al*., 2006; Bakos *et al*., 2011), or semen volume (Qin *et al*., 2007; Jensen *et al*., 2004; Fejes *et al*., 2006; Pauli *et al*., 2008; Magnusdottir *et al*., 2005), other studies have described an association with decreased sperm counts (Jensen *et al*., 2005; Håkonsen *et al*., 2011; Bakos *et al*., 2011; Magnusdottir *et al*., 2011; Braga *et al*., 2012). The conflicting nature of the data published to date hinders the establishment of a clear correlation between having a high BMI and getting pregnant spontaneously or through assisted reproductive technology.

Another important fact is that some of these studies were performed with volunteers (Qin *et al*., 2007; Jensen *et al*., 2004; Aggerholm, 2008; Magnusdottir *et al*., 2005; Gutorova *et al*., 2014), while others were carried out in fertility clinics (Hammoud *et al*., 2008, Chavarro *et al*., 2010; Fejes *et al*., 2006; Pauli *et al*., 2008). This difference may affect the results, since individuals seeking care at a fertility clinic have already tried to conceive without success, while the volunteer group is likely to contain fertile individuals.

The effects of obesity on sperm alterations and male infertility are probably multifactorial. They involve several points, including the hypothalamic-pituitary-gonadal axis and the aromatization of steroids to estrogens in peripheral tissues leading to hyperestrogenic hypogonadotropic hypogonadism (Schneider *et al*., 1979). Associations with altered function of Sertoli cells and decreased spermatogenesis (Winters *et al*., 2006) have been described, in addition to increased scrotal temperature caused by inguinal and pelvic fat accumulation (and scrotal fat deposition) resulting in higher scrotal temperature and altered spermatogenesis (Shafik & Olfat, 1981).

Obesity has also been associated with erectile dysfunction and decreased fertility secondary to fewer sexual intercourses between obese couples. It has been shown that fertility rates tend to improve after weight loss (Hammoud *et al*., 2008; Qin *et al*., 2007).

Environmental factors may also be involved in sperm quality, as obese men exposed to lower temperatures did not show sperm alterations (Gutorova *et al*., 2014).

The systematic reviews and meta-analyses published on the matter have reported conflicting results (Pasquali, 2006; Sermondade *et al*., 2013). Most of them challenge the composition of the studied groups, while only a few studies have described favorable outcome measures. Additionally, the BMI may be an imperfect measure of obesity due to the different body compositions seen in the population (Prentice & Jebb, 2001).

Even though the WHO has named sperm count, morphology, and motility as the main parameters used to assess male fertility, other factors such as DNA fragmentation, which may improve after weight loss, may be involved. (Sermondade *et al*., 2013, Alshahrani *et al*. 2016).

The individuals enrolled in our study had a history of infertility, which made them a better representation of the population in which a relationship between the BMI and infertility could be established. Hormonal parameters were not analyzed, once this study was not designed to find how obesity might impact sperm parameters in the enrolled population, but rather how spermatogenesis was affected.

## CONCLUSIONS

It is yet to be determined whether infertile men should be advised to lose weight in order to improve their sperm parameters. This study failed to show an association between obesity and male fecundity.
